# Dual inhibition of phosphoinositide 3-kinases delta and gamma reduces chronic B cell activation and autoantibody production in a mouse model of lupus

**DOI:** 10.3389/fimmu.2023.1115244

**Published:** 2023-05-10

**Authors:** Folayemi Olayinka-Adefemi, Sen Hou, Aaron J. Marshall

**Affiliations:** Department of Immunology, Max Rady College of Medicine, Rady Faculty of Health Sciences, University of Manitoba, Winnipeg, MB, Canada

**Keywords:** B lymphocytes, autoimmunity, PI3K signaling pathway, antibodies, germinal center, plasma cell

## Abstract

Phosphoinositide 3-kinase delta (PI3Kδ) plays key roles in normal B cell activation and is chronically activated in malignant B cells. Targeting of PI3Kδ using FDA-approved drugs Idelalisib or Umbralisib has shown efficacy in treatment of multiple B cell malignancies. Duvelisib, an inhibitor targeting both PI3Kδ and PI3Kγ (PI3Kδγi) has also been used for treatment of several leukemias and lymphomas and was suggested to offer potential additional benefits in supressing T cell and inflammatory responses. Transcriptomics analyses indicated that while most B cell subsets predominantly express PI3Kδ, plasma cells upregulate PI3Kγ. We thus assessed whether PI3Kδγi treatment can impact chronic B cell activation in the context of an autoantibody-mediated disease. Using the TAPP1^R218L^xTAPP2^R211L^ (TAPP KI) mouse model of lupus-like disease driven by dysregulated PI3K pathway activity, we performed 4 week PI3Kδγi treatments and found significant reduction in CD86+ B cells, germinal center B cells, follicular helper T cells and plasma cells in multiple tissues. This treatment also significantly attenuated the abnormally elevated serum levels of IgG isotypes observed in this model. The profile of autoantibodies generated was markedly altered by PI3Kδγi treatment, with significant reductions in IgM and IgG targeting nuclear antigens, matrix proteins and other autoantigens. Kidney pathology was also impacted, with reduced IgG deposition and glomerulonephritis. These results indicate that dual inhibition of PI3Kδ and PI3Kγ can target autoreactive B cells and may have therapeutic benefits in autoantibody-mediated disease.

## Introduction

Dysregulation in the phosphoinositide 3-kinase (PI3K) signalling pathway has continually been implicated in multiple types of disease such as cancers, immune metabolic diseases and auto-immune conditions (1). This signalling pathway is critical in modulating immune cell functions such as homeostasis, metabolism and proliferation. In lymphocytes, the activation of class 1 PI3Ks is controlled by antigen receptor, co-stimulatory receptors, cytokines and chemokine receptors (2). Of the four class I PI3K enzymes, PI3Kδ and PI3Kγ are selectively expressed in immune cells (3, 4) PI3Kδ has critical functions in B cells as well as T cells (5), whereas the functions of PI3Kγ in T cells, macrophages and neutrophils are most well established (6). We found that chronic lymphocytic leukemia cells upregulate PI3Kγ and this isoform has non-redundant functions in chemokine responses (7).

Autoimmune diseases result from chronic lymphocyte activation targeting self-antigens resulting from disruption of normal mechanisms maintaining immune tolerance (8, 9). There are over 100 auto-immune diseases affecting a wide range of the population (3-5%), and although there is paucity of information on the exact etiological events leading up to the disease, certain factors such as genetics, environment, sex, diet have been attributed as risk factors (10–13). Systemic lupus erythematosus (SLE) is an autoantibody-mediated disease characterized manifested by a broad spectrum of antibodies targeting nuclear proteins and nucleic acids and immune-complex deposition in the kidney (8). The traditional treatment options for autoantibody-mediated conditions such as SLE include immune suppressive drugs (cyclophosphamide) and non-specific anti-inflammatory (corticosteroids) (9). While more specific and effective treatment options are clearly needed, only two new treatments have been approved in the last 60 years: antibodies targeting the B cell growth factor BAFF (Belimumab) (14) or the type 1 interferon receptor (Anifrolumab) (15).

Several lines of evidence implicate the PI3K pathway in autoimmunity. The inhibitory phosphatase PTEN is primarily known as a tumour suppressor, but has also been described as one of the “gatekeepers” of immunological tolerance (16). Reduced expression of PTEN in SLE patient B cells was suggested as one mechanism of PI3K pathway dysregulation and breaking tolerance (17). Another PI phosphatase SHIP is also known to restrain autoreactive B cells, with acute deletion of SHIP leading to development of autoimmune disease in mice (18). Thus, uncontrolled PI3K pathway activity is implicated in both driving malignant B cell proliferation and driving activation of autoreactive B cells. While the specific PI3K isoforms important in the context of autoreactive B cells remain unknown, treatment with a PI3Kδ-specific inhibitor was able to restore B cell tolerance in PTEN +/- x SHIP +/- mice (19).

Once activated, class I PI3Ks phosphorylate inositol lipid headgroups in the plasma membrane leading to the generation of two major phosphoinositide (PI) species PI(3,4,5)P3 and PI(3,4)P2. These PI products are key players that function in the binding and activation of other downstream signaling molecules with a PI-binding motif (20, 21). The tandem PH domain containing proteins TAPP1 and TAPP2 are adaptor proteins recruited to the plasma membrane by binding specifically to PI(3,4)P2 via their C-terminal PH domains (22, 23). In B cells, TAPP adaptor proteins have functions in regulating germinal centre responses and humoral immunity that depend on PI(3,4)P2 binding. This has been demonstrated by introducing point mutations to TAPP1 and 2 genes within their PI-binding regions, thereby compromising their ability to bind PI(3,4)P2 (24). Uncoupling TAPPs from PI(3,4)P2 resulted in dysregulated activation of the kinase Akt, another PI3K-dependant signaling molecule, as well as dysregulated B cell metabolic programming (25). These TAPP mutant mice develop chronic B cell activation and germinal centres, generate autoantibodies and ultimately develop a lupus-like autoimmune disease.

PI3K pathway inhibition with kinase inhibitor drugs has been extensively explored in anti-cancer therapy, based on genetic or regulatory abnormalities leading to constitutive pathway activity in various cancers (26). Currently approved PI3Ki such as Idelalisib and Alpelisib potently inhibit specific class I PI3Ks isoforms (delta and alpha respectively). Duvelisib is another new generation class I PI3Ki that has been developed to exert dual PI3Kδ/γ inhibition in B cell malignancies (7, 27–31). Several studies have found that dual PI3Kδ/γ inhibitor can also have anti-inflammatory activity in mouse models (32, 33); for example, in an arthritis disease model, individual inhibition of PI3Kδ or γ played a subtle role in diminishing joint disease, while the dual inhibition of PI3Kδ/γ was superior in reducing the induction and progression of inflammatory cells and neutrophils into the joints (34). The impact of PI3Kδ/γ inhibition in the context of autoantibody-mediated disease remains to be explored.

Here we sought to assess the potential of current clinical-grade PI3K inhibitors as therapy for SLE by treating TAPP KI mice with the dual PI3Kδ/γ inhibitor Duvelisib. We find that treatment with dual PI3Kδ/γ inhibitor diminished chronic GC and plasma cells as well as autoantibody levels within these mice. These findings indicate that PI3K inhibition pathway is a potential therapeutic target in controlling SLE type autoimmune disease.

## Materials and methods

### Mice

All animal experiments were conducted according to the Canadian Council on Animal Care guidelines under a protocol approved by the University of Manitoba Animal Care Committee. The origin and phenotype of TAPP1^R211L/R218L^ x TAPP2^R218L/R218L^ (TAPP KI) mice used for the study have previously been reported (24, 35). TAPP KI mice contain mutations in the C-terminal PH domains of both TAPP1 and TAPP2 that abrogate their binding to the phosphoinositide PI(3,4)P2 as described. All mice used for the study were females between 8-9 months old housed in the University of Manitoba Central Animal Care Services (CACS), University of Manitoba Winnipeg, Canada.

### *In vivo* PI3Kδ/γ inhibitor (Duvelisib) treatment

TAPP KI mice with established autoantibody- mediated disease were separated randomly into treatment and sham-treatment groups (drug vehicle only). Duvelisib (IPI-145, Selleck Chemicals LLC, TX, USA) was dissolved according to manufacturer’s instruction and administered at 0.1mg/mouse intraperitoneally (i.p) twice daily for 4weeks. Blood was collected for flow cytometry and autoantibody assessment prior to onset of treatment and weekly post-treatment until endpoint.

### Flow cytometry

Blood, spleen, peyer’s patch, bone marrow and mesenteric lymph nodes were collected and processed to single cell suspensions. Cells were prepared for surface staining with the indicated antibodies. To identify the varying B cell responses fluorochrome-conjugated antibodies against B220 PerCP (clone RA3-6B2), CD19 PE/Cyanine7 (clone 6D5), CD86 PE (clone PO3), CD80 APC (clone 16-10A1), GL7 FITC (clone GL7) and CD95 (Fas) PE/Cyanine7 (clone SA367H8) (Biolegend^®^) were used, and CD4 FITC (clone GK1.5), CD3APC-Cy^™^7 (clone 17A2), PECy7 CXCR5 BV421 (clone 2G8) (BDBioscience^™^) and PD1 PE-Cyanine7 (BDBioscience^™^) were used to assess T cell responses. Cells were stained for 30 minutes and then washed before analysis using a BD FACS Canto II instrument (BD Bioscience, San Diego CA). Data analyses were performed using the FlowJo software (BD Bioscience).

### ELISA and auto-antibody analysis

Total antibody levels for IgA, IgM, IgG1, IgG2a, IgG2b and IgG3 in serum were measured by Mesoscale Discovery U-PLEX assay, according to the manufacturer’s instructions. Auto-antibody profiling of over 120 known auto-antibodies was done pre-treatment and at endpoint by protein microarray at the University of Texas, Southwestern Medical Centre Microarray core facility. Net fluorescent intensity (NFI) and signal-to-noise ratio (SNR) values were used to calculate the antibody score which is the log2 transformed (NFI x SNR+1). Anti-nuclear antibodies were also measured using a kit from Alpha Diagnostics International (Catalog number: #5210) and performed according to the manufacturer’s instruction.

### Kidney pathology analysis

Excised kidneys we embedded in OCT compound, snap frozen in liquid nitrogen and 10uM sections cut on a cryostat. After air drying, sections were fixed in 4% paraformaldehyde for 30 minutes at room temperature (RT) and then rinsed before staining. For immunofluorescence staining, sections were blocked with 5% goat serum in PBS for 30 minutes at RT, then incubated with directly-labeled antibodies diluted in TBST+BSA for one hour at RT and rinsed 3 times with PBST. APC-labelled goat anti-mouse IgG was from Jackson ImmunoResearch (cat#115-135-164) and FITC-labeled rat anti-mouse IgM was from BD Bioscience (clone II/41; Cat#553437). Slides were mounted with ProLong gold antifade solution (Invitrogen #P36935) and images captured using an EVOS cell imaging system (ThermoFisher Scientific). Images were analyzed using Fiji software as follows: lines were drawn across the maximum diameter of each individual glomeruli and the Measure function was used to determine the average fluorescence intensity and length (as an estimate of glomeruli size).

### Statistical analysis

Statistical data analysis was carried out using GraphPad prism program (GraphPad Software Inc., CA, USA). Data are presented as means and standard error of mean (SEM). The Student’s t-test were used for comparisons between groups. Figures use the following representation for significance: *p, 0.05, **p, 0.01, and ***p, 0.001.

## Results

### TAPP KI mice show decreased B cell activation following Duvelisib treatment

**Figure 1:**
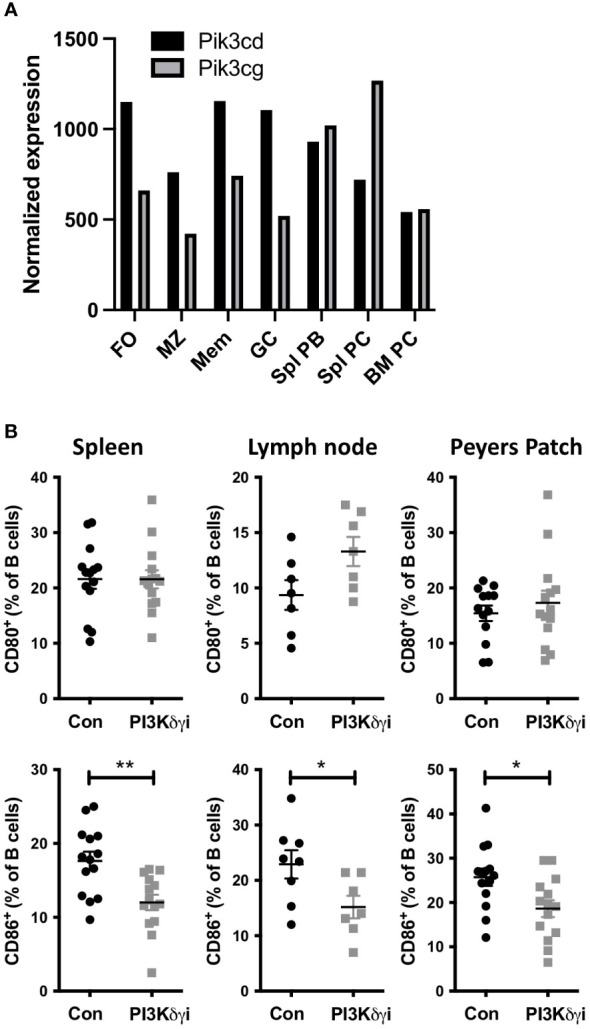
Dual Inhibition of PI3Kδ/γ decreases CD86 expression on B cells. **(A)** Analysis of public RNAseq data showing the differential expression of PI3Kγ (Pik3cg) and PI3Kδ (Pik3cd) catalytic subunits in different B cell subsets. Data were obtained from the Immunological Genome Project (https://www.immgen.org/ImmGenData.html) (36). **(B)** TAPP KI mice were treated for four weeks with dual PI3Kγ and PI3Kδ inhibitor Duvelisib and then sacrificed. Spleen, mesenteric lymph nodes and Peyer’s Patch cells we collected and analyzed by flow cytometry. B cell CD80 and CD86 expression was determined by flow cytometry. Results are pooled from 2 independent experiments with similar results. *, p< 0.05; **, *P< 0.01* by Student’s T-test.

TAPP KI mice develop a lupus-like autoimmunity driven by PI3K pathway dysregulation that is similar in phenotype to other lupus mouse models. While several mouse models of PI3K pathway dysregulation, such as mutation of SHIP, or its binding site in the inhibitory receptor FcγRIIB, result in autoantibody-mediated disease, it is unknown which PI3K isoforms may drive disease. PI3Kδ is generally considered to be the most critical isoform for B cell activation, but PI3Kγ has also been implicated in pathological B cell functions as well as T cell responses. Analysis of public transcriptome data indicates that antibody-secreting plasmablasts and plasma cells in the spleen show increased expression of the PI3Kγ catalytic subunit Pik3cg and lower expression of the PI3Kδ catalytic subunit Pik3cd (**Figure 1A**), indicating that targeting this isoform may also be required to impact autoantibody-mediated disease. Thus, we determined the effect of inhibiting both the PI3Kδ and PI3Kγ isoforms on autoimmune phenotypes by administering dual PI3Kδ/γ inhibitor Duvelisib (PI3Kδγi) to TAPP KI mice with established disease. After daily treatment for 30 days, we assessed the expression of B cell activation markers compared with vehicle-treated littermate controls. We observed that while there was no effect on CD80 expression, PI3Kδγi treatment decreased CD86 expression on B cells from spleen, Peyers patch and lymph node (**Figure 1B**), indicating significant impact on chronic B cell activation observed in this model.

### PI3Kδγi treatment reduces chronic germinal center responses in TAPP KI mice

**Figure 2:**
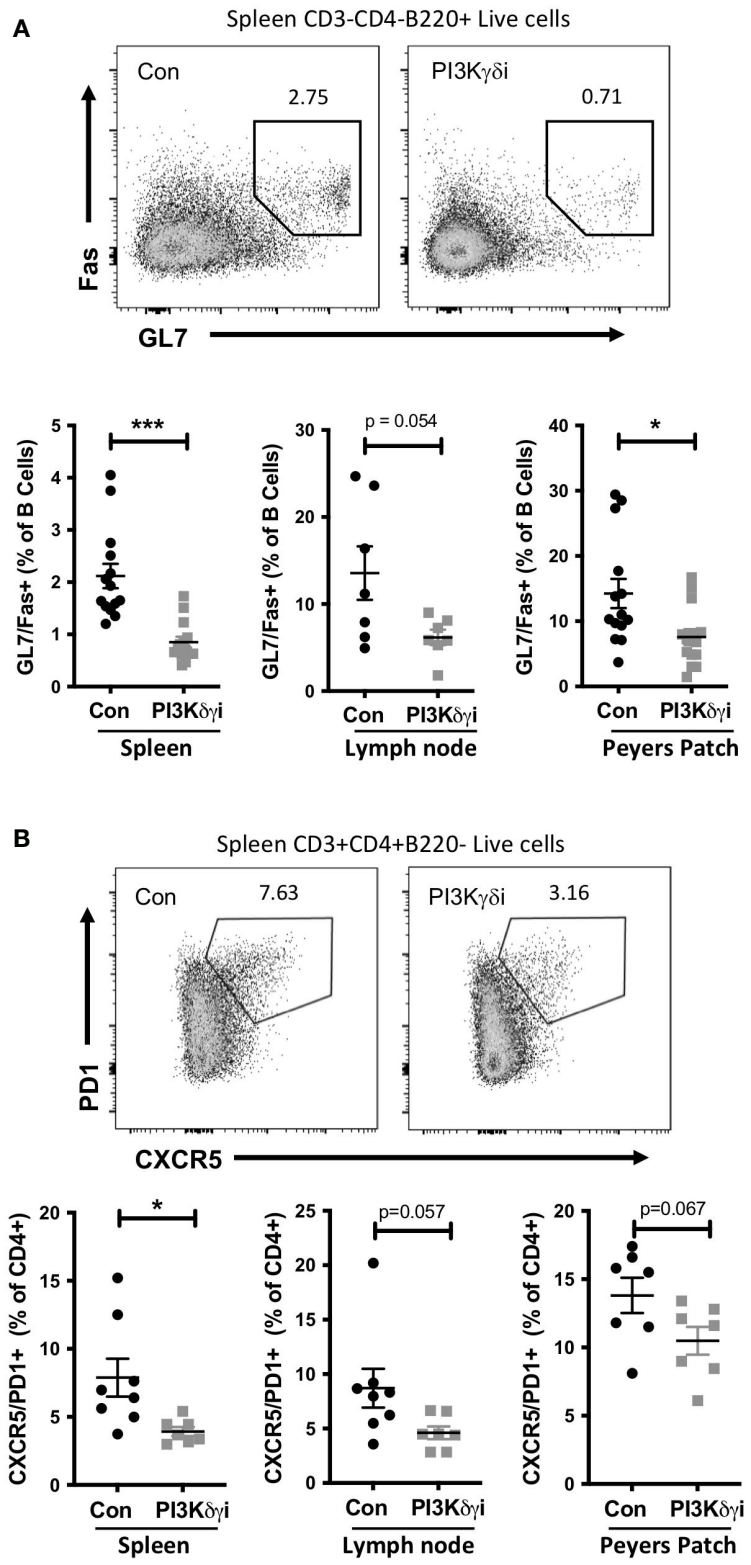
Chronic germinal centre B cell and follicular helper T cell populations in TAPP KI mice are reduced following treatment with PI3Kδ/γ inhibitor. TAPP KI mice were treated for four weeks with Duvelisib and then sacrificed. The indicated tissues were collected and analyzed by flow cytometry. **(A)** Top panels show representative gating of germinal centre B cells (GL7^+^Fas^+^) gated from live B220+ cells. Bottom graphs show results pooled from 2 independent experiments with similar results. **(B)** Top panels show representative gating of follicular helper T cells (CXCR5^+^ PD1^+^) gated from live CD4+ cells. Bottom graphs show results from a representative experiment. *, p< 0.05; ***, P<0.001 by Student’s T-test.

Autoimmune diseases are associated with chronic germinal center (GC)-like structures which persist for months or years and are thought to be important for generation of somatically mutated pathogenic autoantibodies (37). Mouse autoimmune models such as TAPP KI also develop chronic GC with age, and we investigated the effect of PI3Kδγi treatment on these chronic GCs. We found a marked reduction in splenic GC B cells in PI3Kδγi-treated mice compared with the control TAPP KI group (**Figure 2A**). We also noted a decrease in Peyers patch GC B cells, as well a trend of reduced GC B cells in mesenteric lymph nodes of treated mice, suggesting that dual inhibition of the delta and gamma isoforms of PI3K leads to partial dissolution of chronic germinal centers. Since the GC response requires the interaction of the GC B cells with follicular helper T cells (T_FH_) cells (38), we also examined T_FH_ frequencies. A significant reduction in T_FH_ frequencies was observed in spleen, as well as a trend of reduced T_FH_ in mesenteric lymph nodes and Peyer’s patches (**Figure 2B**), indicating a corresponding decrease in these cells as GCs are reduced by PI3Kδγi treatment.

### PI3Kδγi treatment reduces elevated plasma cells and serum antibodies in TAPP KI mice

**Figure 3:**
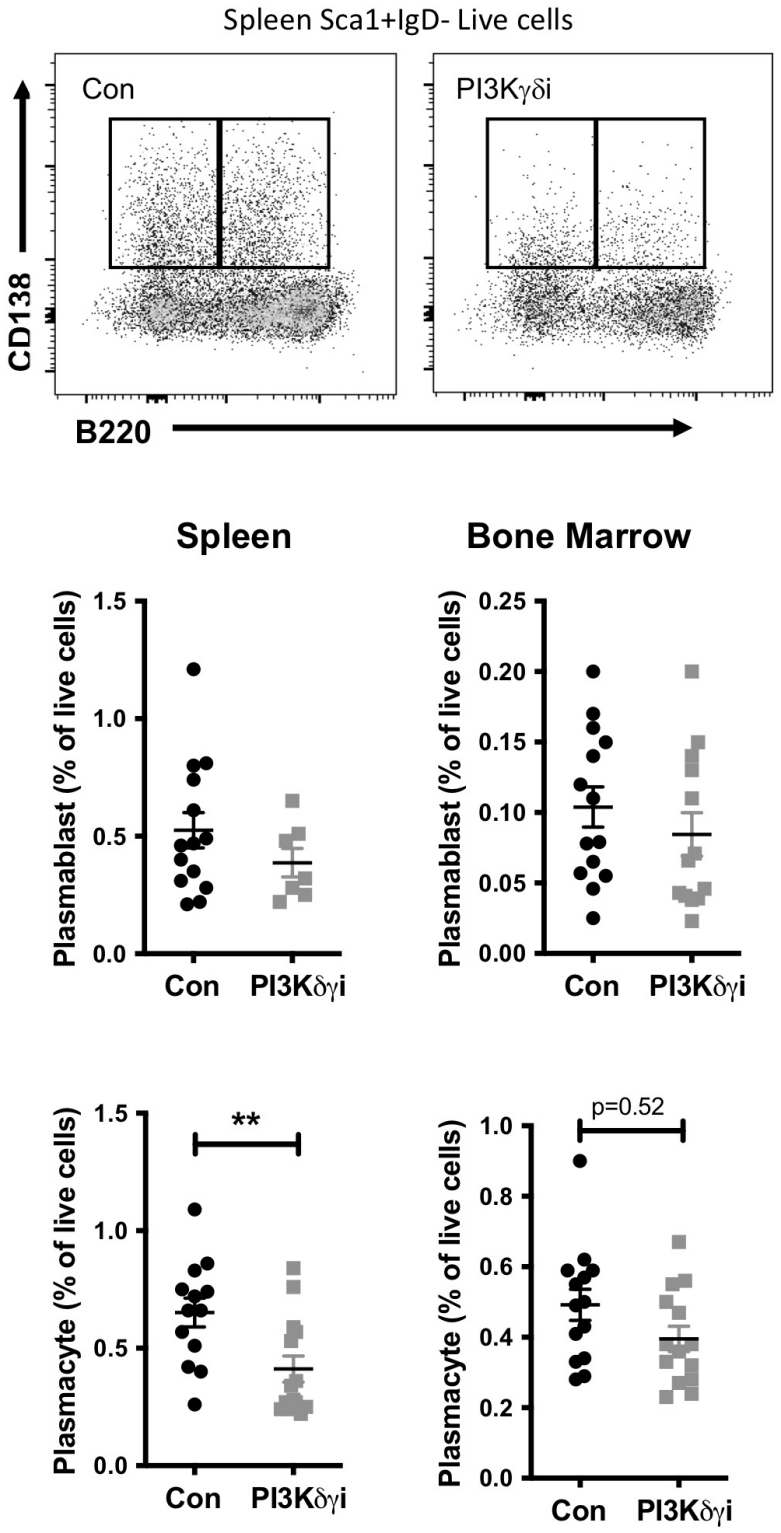
Impact of PI3Kδ/γ inhibition on plasma cells in TAPP KI mice. TAPP KI mice were treated for four weeks with Duvelisib and then sacrificed. The indicated tissues were collected and analyzed by flow cytometry. Top panels show representative gating of plasmablast (CD138+B220+) and plasmacyte (CD138+B220+) cells gated from live Sca1+IgD- cells. Decreased plasmablasts and plasma cells in the spleen and bone marrow of TAPP KI treated mice in comparison to control group by Flow cytometry. Bottom graphs show results pooled from 2 independent experiments with similar results. *, p< 0.05; **, P< 0.01 by Student’s T-test.

**Figure 4:**
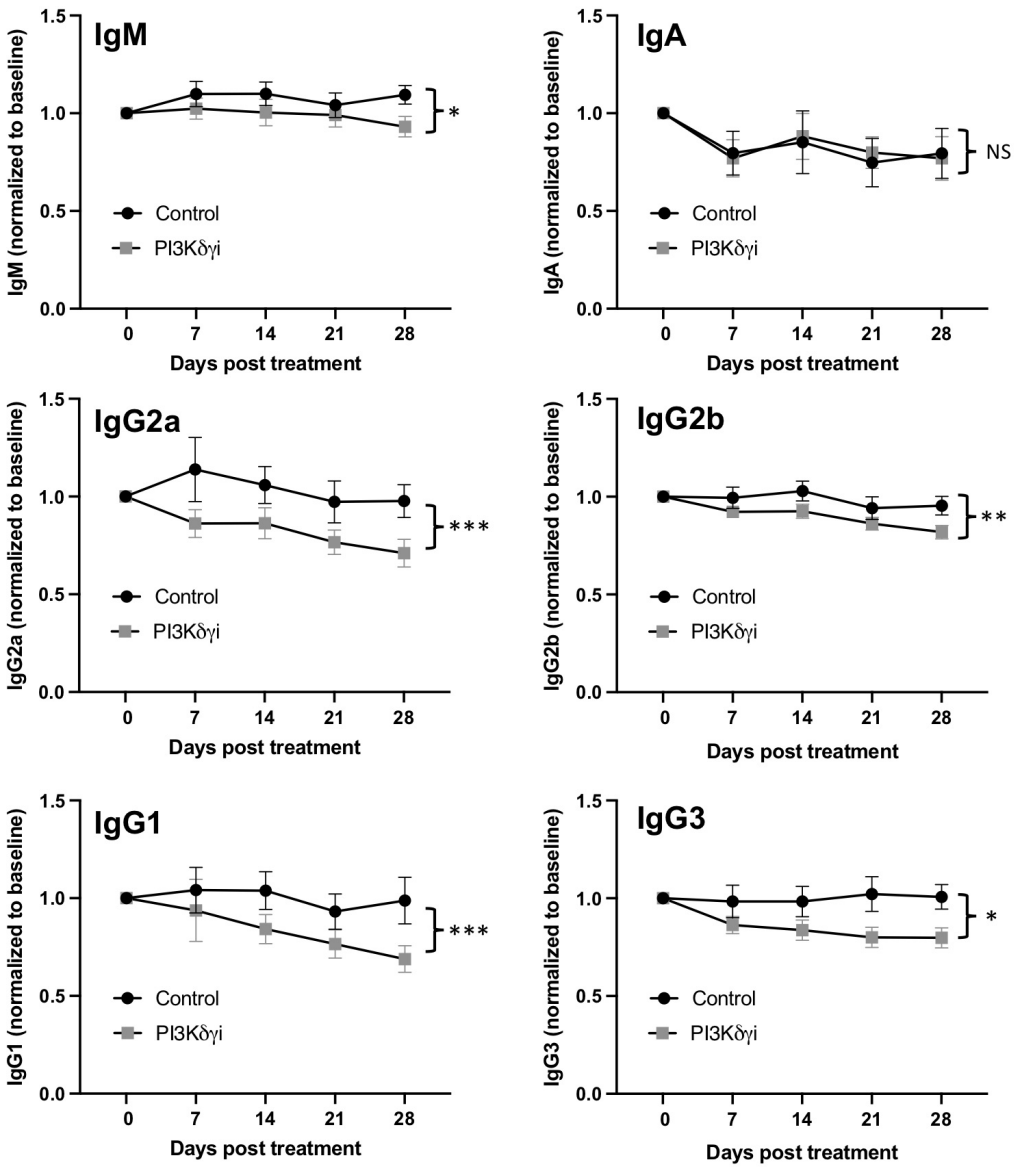
Reduced hypergammaglobulinemia with dual PI3Kδ/γ inhibition. Serum was collected weekly from TAPP KI mice during treatment with Duvelisib. The levels of IgA, IgM, IgG1, IgG2a, IgG2b and IgG3 in TAPP KI mice were then measured by Mesoscale assay. Data were normalized to pre-treatment values for each mouse to determine changes over time after treatment, compared to untreated TAPP KI littermate controls. Results are pooled from 2 independent experiments with similar results (n= 14 mice per group). ns=not significant; *, p< 0.05; **, p< 0.01; ***, p<0.001 by 2-way ANOVA.

Plasma cells are a critical target cell in autoantibody-mediated disease, but also secrete protective natural antibodies and are a key component of humoral memory (39). We found that PI3Kδγi treated mice exhibited a trend toward reduced frequencies of CD138+Sca1+IgD-B220+ plasmablasts in spleen and bone marrow, but this did not reach statistical significance (**Figure 3**). However, frequencies of CD138+Sca1+IgD-B220- plasmacytes were significantly reduced in spleen (p<0.005) and bone marrow (p=0.052) after treatment (**Figure 3**). TAPP KI mice also exhibit hypergammaglobulinemia in conjunction with development of autoimmunity. We therefore examined serum antibody levels within the TAPP KI mice pre- and post-Duvelisib treatment, quantifying total IgA, IgM, IgG1, IgG2a IgG2b and IgG3 (**Figure 4**). Over the four-week treatment period we observed reductions in all antibody isotypes except IgA, with the most significant reductions observed for IgG2a and IgG1. These findings indicate that plasma cells and antibody secretion might be sensitive to therapeutics targeting both PI3Kδ and γ.

### PI3Kδγ inhibitor alters the pathologic auto-antibody profile of TAPP KI mice

**Figure 5:**
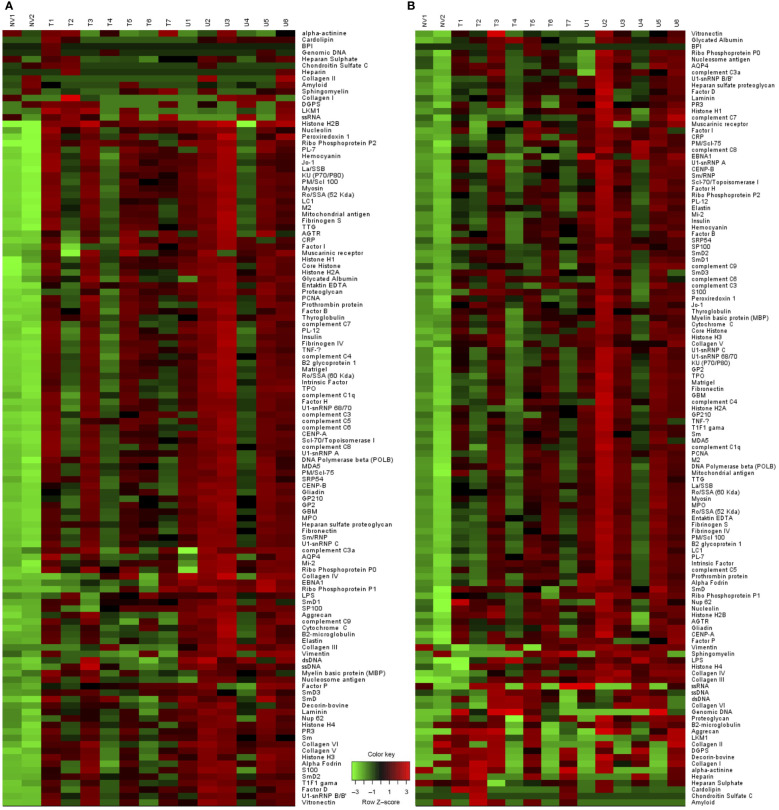
Altered pathologic auto-antibody profile with Duvelisib treatment. Heat map analysis of **(A)** IgM and **(B)** IgG antibodies binding to 120 different autoantigens using proteomic microarray. (NV= Naïve mice, T= Treated, U= Untreated).

**Figure 6:**
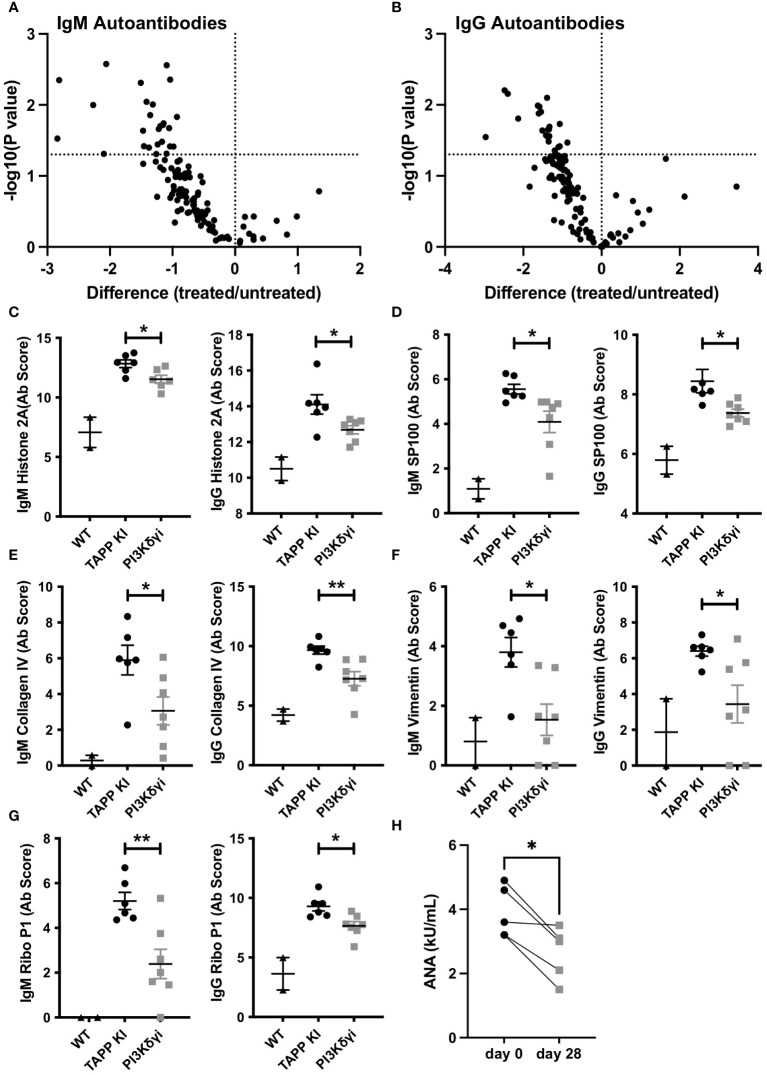
Pharmaceutical inhibition of PI3Kδ/γ reduces auto-antibodies in TAPP mice. **(A)** Volcano plot illustrating significant reductions in specific IgM **(A)** and IgG **(B)** autoantibodies in treated versus untreated mice. **(C-G)** Data for specific significantly reduced autoantibodies. Each data point represents an individual WT C57BL6 mouse, untreated TAPP KI mouse or treated TAPP KI mouse. *, p< 0.05; **, p< 0.01 by Student’s T-test. **(H)** Total anti-nuclear antibody (ANA) levels were assessed pre- and post-treatment using an ELISA-based assay. Pre- and post-treatment values for individual mice are connected by lines. *, p< 0.05 by paired T-test.

Having observed that GC and plasma cells were significantly impacted following inhibition of the PI3K δ and γ, we investigated whether this treatment could impact the profile of autoantibodies produced in this model. An assessment of IgM and IgG antibodies binding to 120 different autoantigens revealed Duvelisib treated TAPP KI mice show generally reduced IgM and IgG autoantibodies compared to mock treated littermate controls (**Figure 5**; **Supp Table 1**). Antibodies against over 20 individual autoantigens were significantly reduced for IgM (**Figure 6A**) and IgG (**Figure 6B**), including antibodies against DNA, nuclear antigens, extracellular matrix components and complement components. For 7 antigens, both IgM and IgG antibodies were significantly reduced by treatment. These included histone H2A (**Figure 6C**), interferon-inducible nuclear antigen SP100 (**Figure 6D**), collagen IV (**Figure 6E**), vimentin (**Figure 6F**) and ribosomal antigen phosphoprotein P1 (**Figure 6G**). We also examined total anti-nuclear antibody (ANA) levels using an ELISA-based assay and found a significant reduction after 4 weeks of treatment (**Figure 6H**). These results indicate that PI3Kδγ inhibition can alter the profile of autoantibodies, including specific reductions in antibodies known to be pathogenic in autoimmune disease.

### Impact of PI3Kδγ inhibitor on antibody deposition in kidney

**Figure 7:**
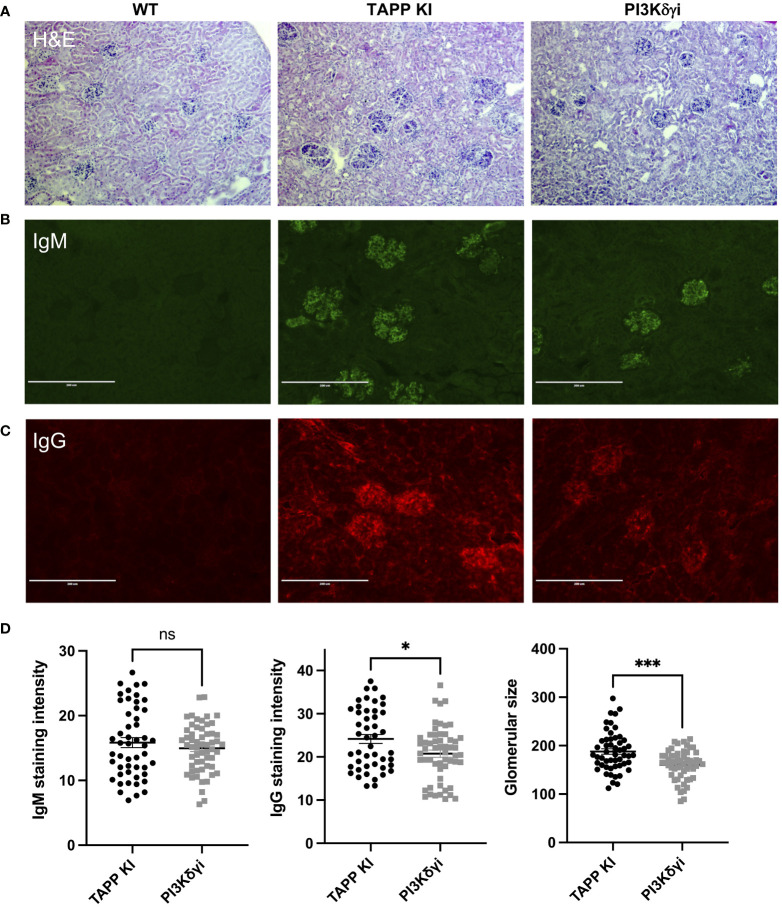
Impact of PI3Kδγ inhibitor on antibody deposition in kidney. **(A)** Kidney sections from the indicated mouse groups were stained with hematoxylin-eosin. Immunofluorescence staining was performed to detect IgM **(B)** or IgG **(C)** deposition. **(D)** Analysis of average IgM or IgG staining intensity across individual glomeruli, as well as maximum glomeruli diameter, was determined using Fiji software. The graph represents data for individual glomeruli (10-15 per mouse) pooled from 4 mice per group, with mean and SEM indicated. *, p< 0.05; ***, p< 0.001 by Student’s T-test.

Our previous studies found that TAPP KI mice exhibit kidney pathology, with antibody deposition and glomerulonephritis; thus we examined whether PI3Kδγ inhibition is sufficient to reverse these hallmark pathologies of lupus. We compared hematoxylin-eosin stained sections from control, TAPP KI or TAPP KI mice treated with PI3Kδγi for 4 weeks (**Figure 7A**). TAPP KI mice showed glomerulonephritis with a variable degree of mesangial proliferation as expected, and post-treatment glomeruli exhibited returned to a more normal histological appearance. Assessment of IgM or IgG deposition by immunofluorescence microscopy (**Figures 7B, C**) staining revealed that treated mice still have obvious antibody deposition in glomeruli, but glomeruli were visibly smaller and more regularly-shaped in the post-treatment group. Digital image analysis indicated that average IgM staining intensity in glomeruli was not reduced after treatment, however IgG staining intensity was reduced (**Figure 7D**). Image analysis also confirmed the significant reduction in average size of glomeruli after treatment (**Figure 7D**). Together these results indicate that four week treatment with PI3Kδγi led to partially improvement of glomerulonephritis in TAPP KI mice.

## Discussion

Novel and innovative strategies for targeting autoantibody-producing B cells in diseases such as SLE are urgently needed. The use of small molecule kinase inhibitors to shut down chronic BCR signaling is a concept that is now well established for treatment of B cell malignancies, with several clinically approved drugs being widely used and more in development. Several lines of evidence suggest that targeting BCR signaling may be of benefit in SLE. A significant fraction of SLE susceptibility genes identified in genome-wide association studies are B cell signaling molecules, indicating that genetic perturbations in B cell signaling pathways can underlie development of SLE (40, 41). Numerous mouse models with disruptions in BCR and co-receptor signaling have been found to lead to development of SLE-like disease pathology (42–44). Finally, as described below a growing number of studies have indicated that treatment of mouse autoimmunity models with pre-clinical kinase inhibitors can reduce disease pathology. Together this evidence suggests that some clinically approved kinase inhibitors initially developed for treatment of B cell malignancies could potentially be repurposed for treatment of autoantibody-mediated disease.

The PI3K pathway is most well-known for its roles in cancer, however multiple lines of evidence have implicated it in autoantibody-mediated disease. Mouse models exhibiting elevated PI3K pathway activity due to deficiency in the activity of PI phosphatases such as SHIP1 or PTEN were found to develop autoantibody-mediated pathology (18, 45). Indeed the key inhibitory receptor on B cells FcγRIIB functions to recruit SHIP (46), and this inhibitory circuit has been implicated in human and mouse SLE studies (47). PTEN has been described as a “gatekeeper” of B cell tolerance, and has been found to be dysregulated in human SLE patients (16, 48). Recent studies found that PI3Kδ gain-of-function mutations led to generation of autoantibodies (49). While most studies indicate that PI3Kδ is the most critical isoform activated by BCR signaling, we chose to examine a dual PI3Kδ/γ inhibitor after noting that plasma cells can downregulate PI3Kδ and increase PI3Kγ expression (**Figure 1**). Another potential benefit of this approach is that both the PI3Kδ and γ isoforms are restricted to hematopoietic cells, unlike PI3Kα inhibitors being actively developed for treatment of solid tumours (50).

In this study we have assessed a PI3K inhibitor drug developed and approved for treatment of B cell leukemia and lymphoma in the TAPP KI mouse model. We previously found that this model exhibits chronic B cell activation and germinal center responses driven by dysregulated PI3K pathway activity (24, 25, 44). Our findings here indicate that the dual PI3Kδ/γ inhibitor Duvelisib can significantly reduce chronic GC and plasma cells responsible for auto-antibody production. The significant reduction of autoantibodies observed within a four-week treatment period is remarkable given that we are reversing established disease and that the half-life of pre-existing IgG antibodies is in the order of weeks. However, in most cases, the reduction was only partial in that it did not reduce autoantibodies to the baseline level of C57BL6 mice. Interestingly, the treatment selectively altered the profile of autoantibodies produced, with the majority showing a trend of reduced levels, but only ~20% being significantly lower. Consistent with a partial reduction in autoantibodies, we found evidence for reduced IgG deposition in kidney and improvement of glomerulonephritis. Improvements in kidney pathology could potentially reflect the effect of PI3Kδ/γi on other elements of the inflammatory process beyond the autoantibodies themselves.

While PI3Kδ/γ inhibition can clearly impact directly on B cells, it is likely to also impact on other cells of the immune system including T cells and myeloid cells. Indeed, we observed reductions in T_FH_ cell populations, which could be due either to direct effects of the treatment on T cells or indirect effects since interactions with GC B cells are known to promote T_FH_ activation and differentiation. Both PI3Kδ and PI3Kγ are expressed and functional in CD4+ T cells and PI3Kδ has been shown to be important for T_FH_ development and function (51, 52). It should be noted that the aim of our study was to assess the potential of repurposing a clinically approved PI3K inhibitor and was not designed to assess B cell-intrinsic versus extrinsic affects.

Other studies have examined the impact of pre-clinical inhibitor compounds selectively targeting PI3Kδ or PI3Kγ on development of various pathologies in mouse models of SLE (53–55); however their impact on B cell subsets and autoantibody profiles were not determined. An inhibitor targeting Bruton’s tyrosine kinase (Btk), whose activity is dependent on binding to PIP3 produced by PI3K, was also found to inhibit generation anti-DNA antibodies in the MRL/Lpr mouse model (56). One study reported that treatment of NZB mice with IPI-145/Duvelisib for 20 weeks reduced anti-DNA antibodies and kidney pathology, consistent with our findings (32). More recently the Cambier group used an elegant model of inducible PI3K pathway dysregulation to acutely reactivate anergic BCR transgenic B cells and demonstrated that even low doses of PI3Kδ-specific inhibitor Idelalisib could prevent generation of autoantibodies in this context (19). Interestingly, reactivation of anergic B cells via deletion of the protein phosphatase SHP1 was not reversed by Idelalisib (19). Thus, our results extend previous findings, which collectively support the concept that dual PI3Kδγ inhibition can reverse autoimmunity driven by PI3K dysregulation.

As an expanding class of small molecule therapeutics, PI3K inhibitors are clearly highly bioactive with broad potential for applications in diseases beyond the initial applications for treatment of B cell malignancies. Notably, inhibitors of class I PI3Ks have been investigated in a number of other autoimmune and inflammatory disorders, including asthma/COPD, collagen-induced arthritis, experimental autoimmune encephalitis and type 1 diabetes (57). While PI3Ki are effective in B cell malignancies, their use has been limited by unexpected toxicities including T cell mediated inflammation (58, 59). Inflammatory toxicities have been largely observed in chronic lymphocytic leukemia patients treated with PI3Kdelta or delta/gamma inhibitors for prolonged periods of time (60, 61). As our study assessed a short-term treatment regimen, we did not assess the development of inflammatory toxicities, which are likely related to impairments in normal regulatory functions of lymphocytes and macrophages (62–64). Thus, a current challenge in developing and repurposing PI3K inhibitors will be to optimize specificity and dosing regimens to maximize benefits while minimizing unwanted effects depending on the disease context.

## Data availability statement

The original contributions presented in the study are included in the article/**Supplementary Material**. Further inquiries can be directed to the corresponding author.

## Ethics statement

The animal study was reviewed and approved by University of Manitoba Animal Care Committee.

## Author contributions

FA and SH carried out experimental work. FA and AM analyzed data. FA and AM wrote the paper. All authors contributed to the article and approved the submitted version.
